# Supplementary figures and images for: A surface pocket in the cytoplasmic domain of the herpes simplex virus fusogen gB controls membrane fusion
Source: PLoS Pathog. 2022 Jun 29;18(6):e1010435. doi: 10.1371/journal.ppat.1010435 (PMC9275723; doi:10.1371/journal.ppat.1010435)

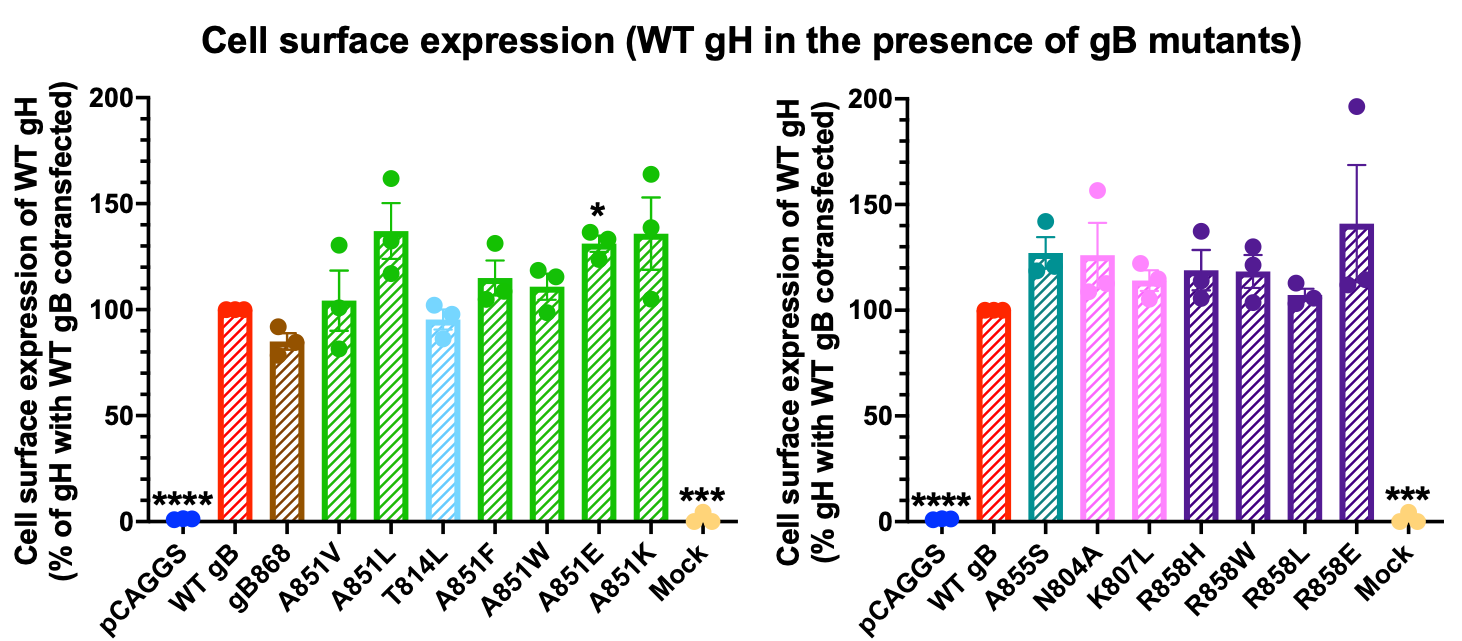

Supplement: S1 Fig — Cell surface expression of gH/gL was tested in the presence of co-transfected gB mutants. LP11 primary antibody. Data are the average of three independent biological replicates. (TIF) [file ppat.1010435.s001.tif]

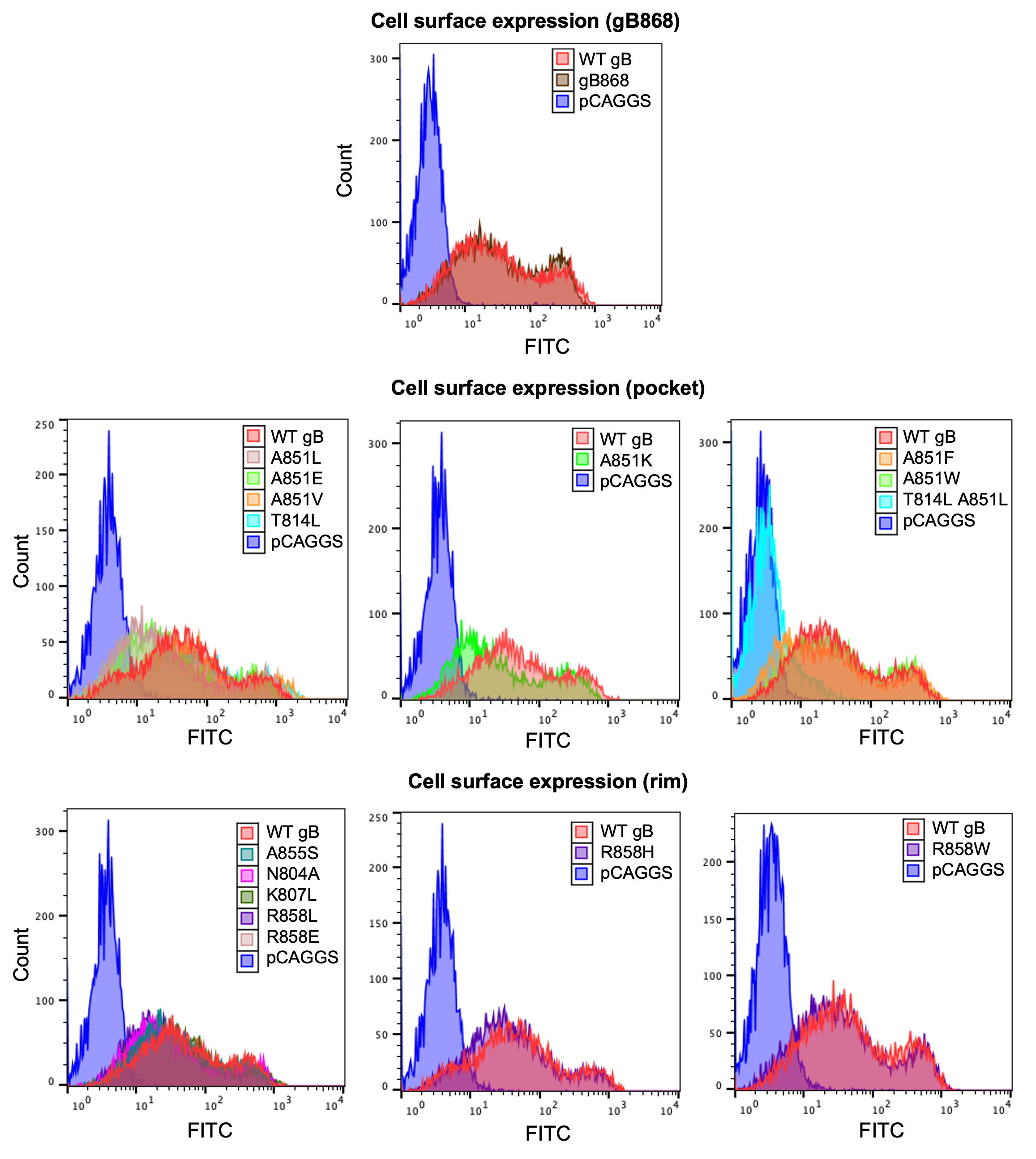

Supplement: S2 Fig — Cell surface expression of gB constructs tested in this work. Mutants tested in separate experiments are shown on separate graphs. The data represent live cells in each condition that were gated using SSC and FSC. FITC signal represents relative levels of gB expression on the cell surface. R68 primary antibody. Data in all panels are from a representative biological replicate. (TIF) [file ppat.1010435.s002.tif]

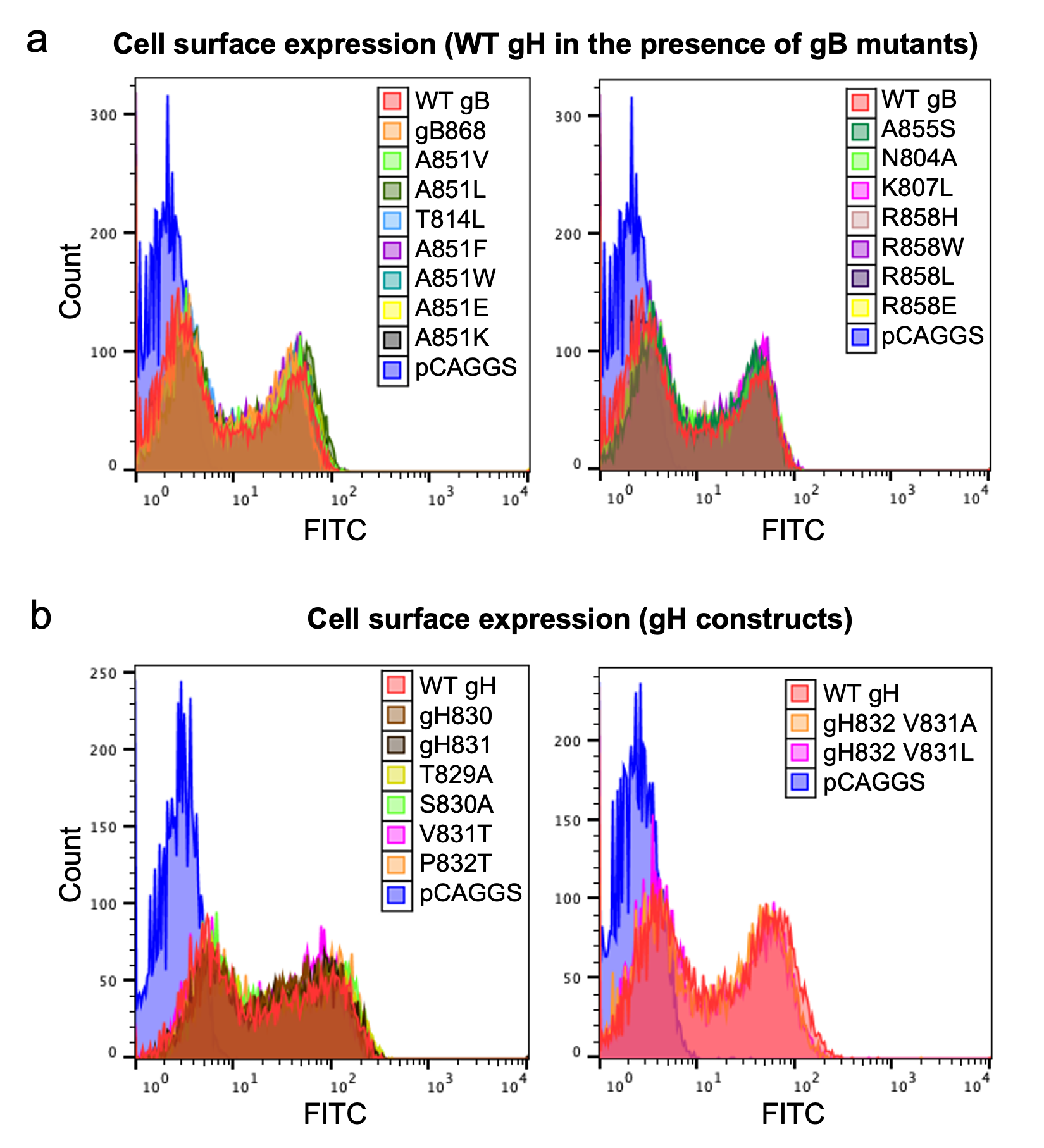

Supplement: S3 Fig — a) Cell surface expression of WT gH/gL in the presence of gB mutants tested in this work. b) Cell surface expression of gH/gL constructs tested in this work. Conditions tested in separate experiments are shown on separate graphs. The data represent live cells in each condition that were gated using SSC and FSC. FITC signal represents relative levels of gH/gL expression on the cell surface. LP11 primary antibody. Data in all panels are from a representative biological replicate. (TIF) [file ppat.1010435.s003.tif]

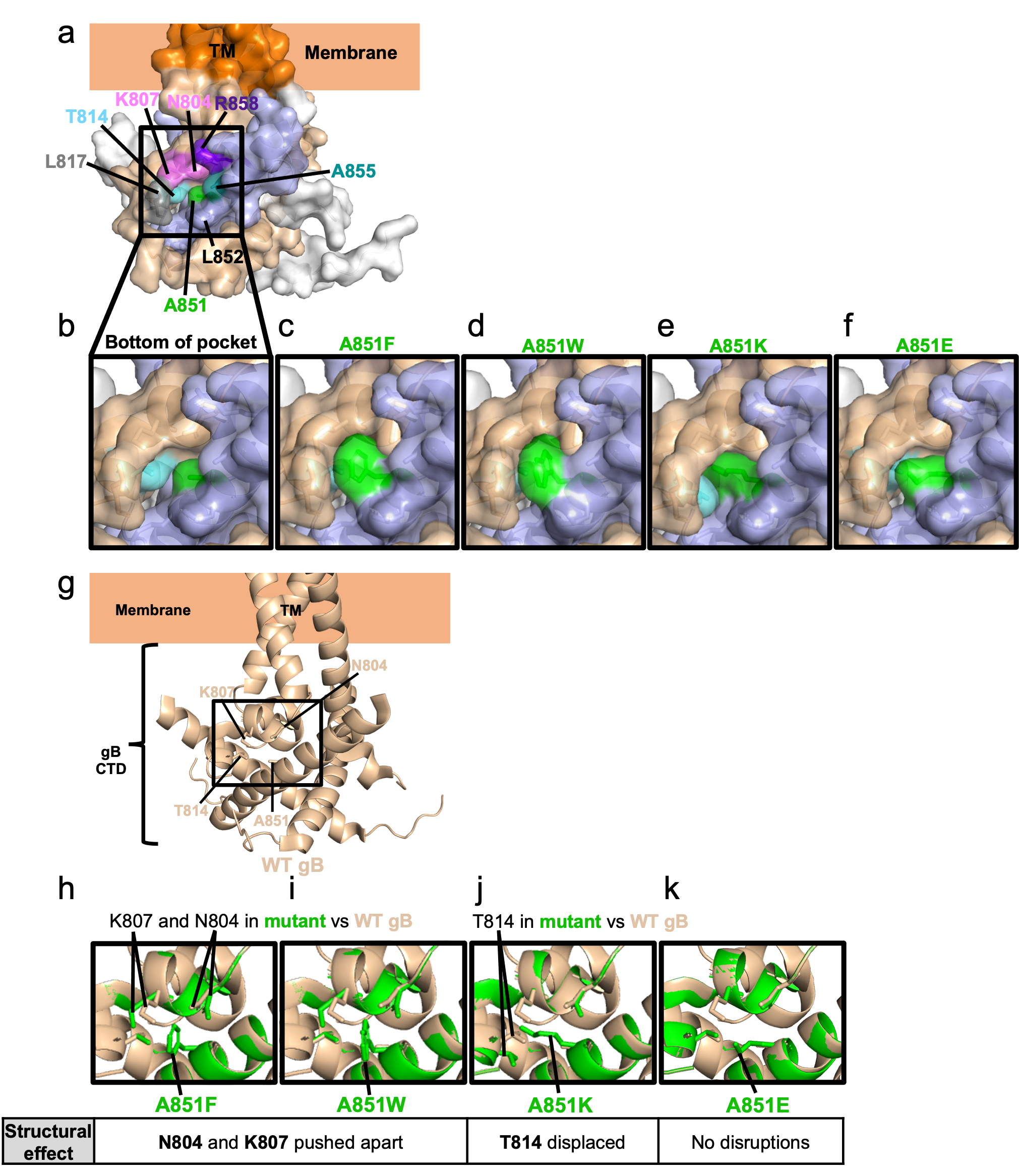

Supplement: S4 Fig — a-b) gBCTD crystal structure and the structure of the pocket on the gBCTD. c-f) gB A851F, A851W, A851K, and A851E mutations were modeled in PyMol and are predicted to fill the pocket. g) WT gBCTD in cartoon representation to visualize predicted effects of mutations on the surrounding structure. h-k) A851F, A851W, A851K, and A851E mutations (green) after energy minimization overlayed onto WT gB (wheat) to show predicted shifts in nearby residues. The large F and W introduced are predicted to cause N804 and K807 to be pushed apart. A851K is predicted to push T814 downwards. A851E is not predicted to cause significant shifts in the nearby residues. (TIF) [file ppat.1010435.s004.tif]
