# Supplementary material for: A surface pocket in the cytoplasmic domain of the herpes simplex virus fusogen gB controls membrane fusion
Source: PLoS Pathog. 2022 Jun 29;18(6):e1010435. doi: 10.1371/journal.ppat.1010435 (PMC9275723; doi:10.1371/journal.ppat.1010435)
Supplement: S1 Table — (DOCX) [file ppat.1010435.s005.docx]

**Supplemental table 1. Primers used for gB_CTD_ mutagenesis.**

| **gB PCR fragments** | **Forward primer**  **Reverse primer** |
| --- | --- |
| A851V piece 1 | 5’-CGACGTGATGGCCGTCTCCACGTGCGTGCCGGTCGCCGCG-3’  5’-CGACACCAGGACCATGTACCGTATCATCTCCCTG-3’ |
| A851V piece 2 | 5’-GGTACATGGTCCTGGTGTCGGCCATGGA-3’  5’-GACGACCTGTGACGGGGGGTTTGTTGTCAATTGCTCTAGCAGATCTTTT-3’ |
| N804A piece 1 | 5’-CGACGTGATGGCCGTCTCCACGTGCGTGCCGGTCGCCGCG-3’  5’-GCCTTCATGGGGGCGCTCTGCAGCCGC-3’ |
| N804A piece 2 | 5’-GCGGCTGCAGAGCGCCCCCATGAAGGC-3’  5’-GACGACCTGTGACGGGGGGTTTGTTGTCAATTGCTCTAGCAGATCTTTT-3’ |
| K807L piece 1 | 5’-CGACGTGATGGCCGTCTCCACGTGCGTGCCGGTCGCCGCG-3’  5’- TACAGGGCCAACATGGGGTTGCTCTGCAG-3’ |
| K807L piece 2 | 5’-AACCCCATGTTGGCCCTGTACCCTCTAAC-3’  5’-GACGACCTGTGACGGGGGGTTTGTTGTCAATTGCTCTAGCAGATCTTTT-3’ |
| T814L piece 1 | 5’-CGACGTGATGGCCGTCTCCACGTGCGTGCCGGTCGCCGCG-3’  5’-AGCTCCTTGAGGGTTAGAGGGTACAGGG-3’ |
| T814L piece 2 | 5’-CCTCTAACCCTCAAGGAGCTCAAGAACC-3’  5’-GACGACCTGTGACGGGGGGTTTGTTGTCAATTGCTCTAGCAGATCTTTT-3’ |
| A851L piece 1 | 5’-CGACGTGATGGCCGTCTCCACGTGCGTGCCGGTCGCCGCG-3’  5’-CGACACCAGGAGCATGTACCGTATCATCTCCCTG-3’ |
| A851L piece 2 | 5’-GGTACATGCTCCTGGTGTCGGCCATGGA-3’  5’-GACGACCTGTGACGGGGGGTTTGTTGTCAATTGCTCTAGCAGATCTTTT-3’ |
| A851K piece 1 | 5’-CGACGTGATGGCCGTCTCCACGTGCGTGCCGGTCGCCGCG-3’  5’-CGACACCAGTTTCATGTACCGTATCATCTCCCTG-3’ |
| A851K piece 2 | 5’-GGTACATGAAACTGGTGTCGGCCATGGA-3’  5’-GACGACCTGTGACGGGGGGTTTGTTGTCAATTGCTCTAGCAGATCTTTT-3’ |
| A855S piece 1 | 5’-CGACGTGATGGCCGTCTCCACGTGCGTGCCGGTCGCCGCG-3’  5’-GCGCTCCATGGACGACACCAGGGC-3’ |
| A855S piece 2 | 5’-CCTGGTGTCGTCCATGGAGCGCACG-3’  5’-GACGACCTGTGACGGGGGGTTTGTTGTCAATTGCTCTAGCAGATCTTTT-3’ |
| R858W piece 1 | 5’-CGACGTGATGGCCGTCTCCACGTGCGTGCCGGTCGCCGCG-3’  5’-TGTGTTCCGTCCACTCCATGGCCGACACC-3’ |
| R858W piece 2 | 5’-CCATGGAGTGGACGGAACACAAGGCC-3’  5’-GACGACCTGTGACGGGGGGTTTGTTGTCAATTGCTCTAGCAGATCTTTT-3’ |
| R858L piece 1 | 5’-CGACGTGATGGCCGTCTCCACGTGCGTGCCGGTCGCCGCG-3’  5’-CTTGTGTTCCGTGAGCTCCATGGCCGAC-3’ |
| R858L piece 2 | 5’-GTCGGCCATGGAGCTCACGGAACACAAG-3’  5’-GACGACCTGTGACGGGGGGTTTGTTGTCAATTGCTCTAGCAGATCTTTT-3’ |
| R858E piece 1 | 5’-CGACGTGATGGCCGTCTCCACGTGCGTGCCGGTCGCCGCG-3’  5’-CTTGTGTTCCGTTTCCTCCATGGCCGAC-3’ |
| R858E piece 2 | 5’-GTCGGCCATGGAGGAAACGGAACACAAG-3’  5’-GACGACCTGTGACGGGGGGTTTGTTGTCAATTGCTCTAGCAGATCTTTT-3’ |
| A851F piece 1 | 5’-CGACGTGATGGCCGTCTCCACGTGCGTGCCGGTCGCCGCG-3’  5’-GGCCGACACCAGGAACATGTACCGTATCATCTCC-3’ |
| A851F piece 2 | 5’-GGTACATGTTCCTGGTGTCGGCCATGG-3’  5’-GACGACCTGTGACGGGGGGTTTGTTGTCAATTGCTCTAGCAGATCTTTT-3’ |
| A851W piece 1 | 5’-CGACGTGATGGCCGTCTCCACGTGCGTGCCGGTCGCCGCG-3’  5’-GGCCGACACCAGCCACATGTACCGTATCATCTCC-3’ |
| A851W piece 2 | 5’-GGTACATGTGGCTGGTGTCGGCCATGG-3’  5’-GACGACCTGTGACGGGGGGTTTGTTGTCAATTGCTCTAGCAGATCTTTT-3’ |
| A851E | 5’-CAGGGAGATGATACGGTACATGGAGCTGGTGTCGGCCATG-3’  5’-GCCTCGGCTAGCTTGGCCTCGTCAAAGTCGC-3’ |
