# Supplementary material for: A surface pocket in the cytoplasmic domain of the herpes simplex virus fusogen gB controls membrane fusion
Source: PLoS Pathog. 2022 Jun 29;18(6):e1010435. doi: 10.1371/journal.ppat.1010435 (PMC9275723; doi:10.1371/journal.ppat.1010435)
Supplement: S2 Table — (DOCX) [file ppat.1010435.s006.docx]

**Supplemental table 2. Primers used for gH_CT_ mutagenesis.**

| **gH PCR fragments** | **Forward primer**  **Reverse primer** |
| --- | --- |
| gH830 piece 1 | 5’-CACGCAGCCCGTGGCCGCAATTGCGCCCGGGTTTCTGGCCGC-3’  5’-GCCCCCTTTAACTTGTCCGGAGAACCTTTAGGATGCCAGCCAGG-3’ |
| gH830 piece 2 | 5’-CCGGACAAGTTAAAGGGGGCGTGGCTTCGGCCGTTTC-3’  5’-CTCTAGAGTCGACCTGCAGGCATGCCTCGAGCTAGCAGATCTTTTTCC-3’ |
| gH831 piece 1 | 5’-CACGCAGCCCGTGGCCGCAATTGCGCCCGGGTTTCTGGCCGC-3’  5’-GCCCCCTTTAGACACTTGTCCGGAGAACCTTTAGGATGCCAGCC-3’ |
| gH831 piece 2 | 5’-GACAAGTGTCTAAAGGGGGCGTGGCTTCGGCCGTTTC-3’  5’-CTCTAGAGTCGACCTGCAGGCATGCCTCGAGCTAGCAGATCTTTTTCC-3’ |
| T829A piece 1 | 5’-CACGCAGCCCGTGGCCGCAATTGCGCCCGGGTTTCTGGCCGC-3’  5’-TCCAAAAAAACGGGACACTTGCCCGGAGAACCTTTAGG-3’ |
| T829A piece 2 | 5’-GTTCTCCGGGCAAGTGTCCCGTTTTTTTGGAG-3’  5’-CTCTAGAGTCGACCTGCAGGCATGCCTCGAGCTAGCAGATCTTTTTCC-3’ |
| S830A piece 1 | 5’-CACGCAGCCCGTGGCCGCAATTGCGCCCGGGTTTCTGGCCGC-3’  5’-TCCAAAAAAACGGGACAGCTGTCCGGAGAACCTTTAGG-3’ |
| S830A piece 2 | 5’-GGTTCTCCGGACAGCTGTCCCGTTTTTTTGGAG-3’  5’-CTCTAGAGTCGACCTGCAGGCATGCCTCGAGCTAGCAGATCTTTTTCC-3’ |
| V831T piece 1 | 5’-CACGCAGCCCGTGGCCGCAATTGCGCCCGGGTTTCTGGCCGC-3’  5’-GCGTCTCCAAAAAAACGGGGTACTTGTCCGGAGAACC-3’ |
| V831T piece 2 | 5’-GGACAAGTACCCCGTTTTTTTGGAGACGCG-3’  5’-CTCTAGAGTCGACCTGCAGGCATGCCTCGAGCTAGCAGATCTTTTTCC-3’ |
| P832T piece 1 | 5’-CACGCAGCCCGTGGCCGCAATTGCGCCCGGGTTTCTGGCCGC-3’  5’-GCGTCTCCAAAAAAACGTGACACTTGTCCGGAGAACC-3’ |
| P832T piece 2 | 5’-CCGGACAAGTGTCACGTTTTTTTGGAGACG-3’  5’-CTCTAGAGTCGACCTGCAGGCATGCCTCGAGCTAGCAGATCTTTTTCC-3’ |
| gH832 V831L insert | 5’-CACGCAGCCCGTGGCCGCAATTGCGCCCGGGTTTCTGGCCGC-3’  5’-GGAAAAAGATCTGCTAGCTCGAGCATGCCTACGGGAGACTTGTCCGGAGAACC-3’ |
