# Supplementary material for: A surface pocket in the cytoplasmic domain of the herpes simplex virus fusogen gB controls membrane fusion
Source: PLoS Pathog. 2022 Jun 29;18(6):e1010435. doi: 10.1371/journal.ppat.1010435 (PMC9275723; doi:10.1371/journal.ppat.1010435)
Supplement: Attachment — Submitted filename: Pataki-PLOSPath-response-to-criticisms.docx [file ppat.1010435.s007.docx]

**Ref: PPATHOGENS-D-22-00454**

**A surface pocket in the cytoplasmic domain of the herpes simplex virus fusogen gB controls membrane fusion**

We appreciate the reviewers' praise for the significance of our findings, good experimental design, and the well written manuscript. We also appreciate their thoughtful criticisms and the opportunity to submit the revised version of the manuscript. Our point-by-point response is provided below. For convenience, we provide both the “clean” and the tracked versions of the revised manuscript.

**Reviewer Comments:**

**Reviewer 1**

**Summary**

This mutational analysis of HSV entry proteins B and H determined regions of the cytoplasmic tails of these proteins involved in regulating membrane fusion. A panel of mutants were tested in a split-luciferase CHO cell fusion assay to determine fusion activity relative to WT. The level of surface expression was also determined to rule out major effects of mutations on protein folding and surface-targeting. A structural pocket was identified on gBCTD. Mutations of residues inside the pocket tend to reduce fusion, while mutations at the rim of the pocket tend to enhance fusion. On the gH side, residue V831 appears to play a critical role in fusion. Using structural modeling, the authors propose that gH V831 inserts into the gB pocket to act as a wedge and dislocates the gB trimer, thereby allowing conformational changes required for gB fusogenic activity. The model is attractive and consistent with the data. The main weakness of the data resides in the lack of statistical significance of many mutations. Despite this, many non-statistically significant trends of several mutants are heavily incorporated to support the model. The authors acknowledge this issue and the model stands based only on the statistically significant data. This statistical issue seems likely caused by the limited number of experimental repeats (3) for an experiment known to be highly variable in efficiency (transient transfections). The authors must thoroughly revise their manuscript to avoid all overinterpretation or add to the data for trends to reach statistical significance. The study is well designed, and the question addressed is important in the field of herpesvirus entry. The authors also suggest that this gH-gB activation model could be used by other herpesviruses. The relevance of this study would also be strengthened by verifying the conservation of the involved gH residues and gB pocket structure in other viruses. The manuscript is well written, and the well-designed experiments have the appropriate controls.

**Major Issues: Key Experiments Required for Acceptance**

1) The main issue with the data are the statistics. This led to contradictory statements such as “R858W and R858L were markedly hyperfusogenic, judging by the mean value, even though increased fusion levels of R858W and R858L were not statistically significant, due to large SEM” (page 10, lines 10-12). Transient transfection experiments are notoriously variable in efficacy, resulting in larger SEM. Most experiments are repeated three times, which for such experiments limits the ability of repeated data to reach significance. Even positive hyperfusogenic controls, fail to reach significance (figure 2). It is likely that repeating the experiments a couple of time more may allow more differences to reach statistical significance. To their credit, for the most part, the authors acknowledge this problem, but unfortunately it does not mean statistics can be ignored in global conclusions. This being said, the body of data support the role of the gBCTD pocket in regulating fusion, but stronger statistical significances would certainly help support this model.

**Response:** We agree and have repeated the experiments referenced by the reviewer three more times. Additional data points have been included in Figs. 2 and 3. Upon the addition of three more replicates, the fusion of gB868 and R858W have become statistically significant. Mutants that were not statistically significant previously, have remained that way. Mutants that we reported as hyperfusogenic but were not statistically significant (N804A, K807L, R858L, A851E) are now described as ‘fusion-neutral’, and interpretations about their effects on fusion have been adjusted accordingly, on lines 482-486.

2) P6 L13-18: It is surprising than the hyperfusogenic control gB868 is not more significantly different than WT gB. Based on statistics in figure 2, only the early extent of fusion is mildly significantly higher than WT gB. Therefore, this sentence in the text is incorrect. However, gB868 has widely been recognised as hyperfusogenic based on less detailed assays.

P10 L21 “the mutations of the rim of gBCTD are overwhelmingly fusogenic with the exception of R858E.” In fact, only two of the seven mutations are significantly hyperfusogenic. This is not overwhelming.

P16 L24: R858E was the only rim mutation that was fusion-neutral. This statement is incorrect since 5 constructs, not only R858E, are not statistically significantly different from WT in fusion assay (figure 3h).

**Response:** With additional biological replicates, gB868 is statistically hyperfusogenic in all measured parameters. Some of the rim mutants did not become statistically significant with the additional replicates. Therefore, we have changed the above statements to refer to the rim mutants as either hyperfusogenic or fusion-neutral on lines 247, 260-262, and 469-470. We have updated Figs. 2 and 3 with the additional data.

3) Flow cytometry quantification. The quantification of cell surface expression by flow cytometry appears to account for the percentage of transfected cells as well as the level of expression of each transfected cell (product of %transfected and geometric mean of fluorescence). It is unclear what impact the efficiency of transfection has on this quantification. HSV protein surface expression has customarily been tested by cell-based ELISA, which tacitly implies equivalent transfection efficacy of the different plasmids. Do the authors obtain the same significance by simply comparing the mean fluorescence of the SSC/FSC-gated population? Presentation of the FACS profiles would greatly help compare WT gB with mutant gB surface expression.

**Response:** We agree that the efficiency of transfection can also cause variability in the flow cytometry results. Lower transfection efficiency will result in fewer gB+ cells and lower final values due to the multiplication of MFI by the % of gB+ cells. We have generated new Supplemental Figs. 2 and 3, in which we show the cell surface expression data as flow cytometry profiles. Based on our data, our interpretation is that transfection efficiencies are high and are relatively consistent among experiments (e.g., compare WT gB profiles across experiments). Therefore, we do not think that variability in transfection efficiency is a significant concern or that it would change the interpretation of our results. We prefer to keep the figures with bar graph quantification in the main text. We believe the advantages of this representation are that it is quantitative, clearly shows multiple biological replicates, allows for easier comparisons of the multiple mutants, and shows statistical comparisons.

Upon reviewer’s request, we re-analyzed the flow cytometry data by taking the MFI of the entire SSC/FSC-gated population. Virtually identical results were obtained with the two methods of analysis (pocket mutants shown below as an example). The difference between the two methods is that in our method, all gB- cells are treated the same, while in the reviewer’s suggestion, gB- cells will have variable amounts of fluorescence, which affects the overall MFI and can slightly increase the variability. For example, if 10% of the cells are gB-, the MFI is multiplied by 0.9 (the fraction of gB+), regardless of whether all the gB- cells have zero fluorescence or higher fluorescence (but still below the gB+ cutoff) or somewhere in between. In the reviewer’s suggestion, the distribution of the gB- cells can reduce the overall MFI to a greater or lesser extent, depending on how the gB- cells are distributed. However, in our case the effect of the different methods of analysis on our results turned out to be minimal.

**(alternative analysis)**

**(from manuscript)**

4) P2 L12-15, P5 L20-22: The authors suggest the interaction of gHCT with the gB pocket described for HSV-1 could be used by other homologs because gB and gH are conserved among herpesviruses. This is interesting but not explored in this study. To maintain this statement in the abstract, it would be worth to analyse specifically the sequences of gHCT homologs to identify if V831 is conserved (or replaced by a residue with similar properties). Similar analysis could be done with gBCTD sequences. In addition, current advances of 3D modeling could also help model gBCTD from other viruses to determine if the pocket structure is maintained. These comparisons would reinforce the relevance of the mechanism presented here for HSV-1.

**Response:** We thank the reviewer for this excellent suggestion and have included the alignments of gB_CTD_ and gH_CT_ sequences of several herpesviruses in a new figure, Fig. 6. We have also included a new section in the Discussion describing the predicted conservation of the gB_CTD_ pocket and rim, gH_CT_ wedge, and triggering mechanism across herpesviruses on lines 515-549.

5) P17 L5: The reason for the apparent absence of phenotype of pocket-filling mutation is that they also push protomers apart, thereby being more likely to separate and be more fusogenic. If that’s the case, wouldn’t we expect these mutants to have exhibit some gH-independent fusion, if these mutations by themselves do what gH V831 is supposed to do? Could the author elaborate in this paragraph?

**Response:** Thank you for raising this important point. We and others have shown that the hyperfusogenic mutants of HSV-1 gB still require gH/gL, which suggests that these mutations weaken the gB_CTD_ clamp but do not abolish it. We have added additional explanation in that paragraph, lines 503-507, to clarify that we hypothesize that the A851F, A851W, A851E, and A851K mutants act similarly to other hyperfusogenic mutants containing mutations predicted to destabilize the clamp.

6) Fig 2a: for completion, gB868 could be included. This is also relevant given the lack of statistical difference in hyperfusogenicity from several parameters detailed in this figure.

**Response:** We have added cell surface expression level of gB868 to Fig. 2a.

**Minor Issues: Editorial and Data Presentation Modifications**

7) P2 L9: the meaning of “fault line” is unclear. The part of the sentence with this jargon may not be necessary in the abstract. However, it should be explained in the text P5 L13. Explanation is provided later (P10 L17). In the abstract, “fault line” could be replaced by protomeric interface.

**Response:** We agree and have modified the text on lines 27-28 to read “The gB pocket is located above the interface between adjacent protomers…”. The text on lines 124-125 and 447-448 has been modified to read “The gB pocket is located above the interface between adjacent protomers, which is akin to a “fault line” within the gB_CTD_ trimer”.

8) P3 L19: spell out vesicular stomatitis virus.

**Response:** We have made this correction on line 67.

9) P3 L28-29: This sentence needs to be rephrased for clarity. Indirect interactions with receptor is a bit confusing. It also suggest that gH function is to bind receptors.

**Response:** We have rephrased this sentence to clarify that gH/gL binds receptor-binding viral proteins, lines 75-77.

10) P11 L17-18: this is slightly overinterpreted. This amino acid is the most important among those tested. Same comment in page 14, line 19.

**Response:** We agree and have changed the text to read “Collectively, these data implicated V831 as the most important gH_CT_ residue for fusion among those tested.” on lines 315-316 and “Using mutational analysis, we identified gH_CT_ residue V831 as the single most critical residue for fusion among those tested.” on lines 438-439.

11) P13 L19: Since the structure is based on a model: “without otherwise presumably perturbing the surrounding gBCTD structure”

**Response:** We have changed the text accordingly on lines 405-408.

12) Figure 1: it should be mentioned that glycoprotein structures are not drawn to scale (a gD monomer is much smaller compared to gH or gB)

**Response:** We apologize for this omission and now show all structures in Fig. 1 on the same scale.

13) Legend figure 2 (line 3): PDB numbers and citations are not relevant in this legend and should be replaced by glycoprotein names. “an HSV receptor (3U83 [29])” should be “nectin-1”

**Response:** We have replaced “an HSV-1 receptor” with “nectin-1 receptor”, and we have added the names of the glycoproteins. We prefer to keep the PDB numbers and citations to make it clear which structures were used in making the figure.

14) Figure 3h,i: any significance of the colours of the bars (mostly pink vs violet)

**Response:** The colors of the bars were chosen to match the colors of the residues in the crystal structure as shown in Fig. 3a.

15) Figure 4g: the colour of some value dots (pink) do not match the colour of the bars (blue and green). It may be on purpose, if so, then the left part of the panel should have red dots.

**Response:** This was done intentionally to indicate that combinations of gB and gH mutants are being tested. Red dots have been added as suggested.

16) Reference 13: typographic error in title

**Response:** We have corrected the error.

**Reviewer 2**

**Summary**

In this manuscript, Pataki et al. investigated the mechanism of how HSV-1 gH triggers gB fusogenic activity by using a split-luciferase cell-cell fusion assay. They demonstrated that a surface pocket within the gB CTD and residue V831 within the gH cytoplasmic tail are each important to trigger cell-cell fusion. Overall, this is an important finding which will deepen the knowledge of HSV-1 membrane fusion mechanism. However, the methodology used to perform and evaluated the mutant prediction model is incompletely described, which lowers the confidence of their overall model. They should address following major comments for publication.

**Major Issues: Key Experiments Required for Acceptance**

1. For Fig 2, please show gH surface expression, to rule out the possibility that gB mutants (eg. A851V) do not disturb gH expression/folding. Same for Fig. 4.

**Response:** The reviewer must be referring to Figs. 2 and 3. We conducted additional experiments testing the effect of gB mutants on gH/gL cell surface expression and have included these results in the new Supplemental Figs. 1 and 3a.

2. Additional information should be provided for how the modeling was done. Please include in the manuscript the prediction models for gB A851F, A851W, and A851K. Please include information on how the accuracy of their predicted structure models was done. Is there a statistical analysis to show that their chosen models has the highest confidence?

**Response:** We agree and have added a more detailed description of the modeling strategy to the Methods. Most common rotamers were selected while keeping clashing and strain to a minimum. Additional details have been added to the explanation on lines 676-689. We are not aware of calculations or statistical tests that can determine the probability that the chosen rotamers and overall structures are accurate. Doing so would require experimental structure determination.

We now illustrate the predicted structural effects of the A851F, A851W, A851K, and A851E mutants in the new Supplemental Fig. 4. This includes the predicted structural effects on the gB_CTD_ pocket as well as predicted structural disruptions to the nearby area.

3. The evidence provided is not sufficient to support the model presented in figure 5.

-Please describe details on how the distances from the gB CTD pocket or gH V831 to the membrane were estimated.

**Response:** The first residue of the gH_CT_ (modelled as an unstructured peptide in PyMol) was positioned manually in PyMol at the TMD-CTD border of the gB crystal structure so that the cytoplasmic domain of gB and gH would start at the same point. In this position, the gB pocket and gH V831 overlapped visually, as shown in Fig. 5a, meaning that they are the same distance from the membrane by this analysis. This explanation has been added on lines 689-698.

- Please provide the experimental evidence that the 14aa gHct has an extended conformation.

**Response:** We do not know of an experiment to determine the gH_CT_ conformation. Nevertheless, we hypothesize that the gH_CT_ has an extended conformation because it is predicted to be unstructured. Moreover, the gH_CT_ is relatively short and contains residues that are disfavored in α-helices, such as proline and valine. We have added this explanation on lines 317-318.

-Although they have pointed out that they don’t have the direct evidence of the interaction between the gB pocket and the gH V831 interaction, the reviewer still thinks this is a very important piece of data to support their model. Therefore, they should provide direct evidence of gH pocket and gH V831 interaction. They could potentially perform a GST-Pulldown assay or co-IP assay using their mutant set.

**Response:** The reviewer brings up an important point. Whether gH V831 interacts with the gB_CTD_ pocket directly is a key question. However, answering it is challenging because gH and gB interact through multiple domains, which we have shown in a recent study that is currently under peer review and has been uploaded to BioRxiv (doi: https://doi.org/10.1101/2022.05.07.491029). Specifically, we found that gH and gB interact through their ectodomains, transmembrane domains, and cytoplasmic domains, although the respective binding sites have not yet been mapped. Moreover, we found that disrupting interactions between the gH and gB cytoplasmic domains did not reduce overall gH-gB interaction, presumably, due to the remaining interactions mediated by the ectodomains and transmembrane regions. We have added text in the Discussion on lines 555-560 to highlight these findings.

We respectfully disagree that a GST-pulldown assay or co-IP would provide direct evidence of the interaction between the gB pocket and the gH V831. First, neither a GST-pulldown assay nor a co-IP would establish that these proteins interact directly. Furthermore, perturbing the interactions between gH V831 and the gB_CTD_ pocket is unlikely to disrupt the interactions between gH and gB due to remaining interactions between the ectodomains and transmembrane regions. Finally, we hypothesize that the gH_CT_ likely has several points of contact with the gB_CTD_ that help properly orient gH V831 in relation to the pocket prior to wedging. Therefore, we think that the gH_CT_ is unlikely to interact with the gB_CTD_ using gH V831 alone. Detection of direct interactions between gH V831 and the gB_CTD_ pocket is the subject of future work, beyond the scope of this manuscript.

- Please provide the experimental evidence that gH V831 side chain can destabilizes the gB CTD inhibitory clamp. They could potentially perform a FRET assay to detect the conformational changes of gB CTD inhibitory clamp upon gH V831 insertion.

**Response:** Whether gH V831 destabilizes the gB_CTD_ inhibitory clamp and how it does so is an important question that we would like to answer. However, it is a challenging endeavor, and, at the moment, we cannot think of an experiment that could be done in the near future to answer this question. The proposed gB_CTD_ destabilization is likely only temporary because it is a structural transition state between the gB prefusion and postfusion conformations. We hypothesize that the gB_CTD_ destabilization allows the gB ectodomain to rapidly refold, while the gB_CTD_ snaps back into its original conformation. We also hypothesize that the conformational change that occurs within the gB_CTD_ is relatively minor, with gH V831 causing only a slight separation between protomers that momentarily releases the clamp, as opposed to major refolding events that occur in the gB ectodomain.

We thank the reviewer for the idea of a FRET assay. Developing a FRET assay to test this would require extensive work and optimization. In addition, we doubt that a FRET assay would be able to reliably detect a potentially small, temporary conformational change and generate fluorescence significantly above background levels. Detection of conformational changes in gB_CTD_ in response to gH is the subject of future work, beyond the scope of this manuscript.

**Minor Issues: Editorial and Data Presentation Modifications**

1. Please comment on whether gH V831 and gB T814/A851 are conserved in homologous gH or gB of other herpesviruses?

**Response:** We have added a new figure, Fig. 6, to illustrate the conservation of the gH V831 and gB_CTD_ pocket across herpesviruses. We have also added a description in the Discussion on lines 515-549.

2. Please remove description of results from the figure legends and instead just focus on key details required to understand each figure.

**Response:** We have modified the legend of Fig. 2 accordingly.

**Reviewer 3**

**Summary**

Herpesvirus entry requires multiple glycoproteins. gH/gL and gB are components of the entry machinery that are conserved in all herpesviruses. gH/gL triggers the viral fusogen gB to mediate fusion; however, the mechanism of gH/gL signaling is unknown. This work addresses this critical gap in knowledge, using structural models and mutagenesis to identify specific sites in both the gB and gH cytoplasmic tails that may interact to trigger gB. The resulting proposed model is that gH tail residue V831 interacts with a pocket in the gB tail (between residues T814 and A851), acting as a wedge that separates the gB protomers and triggers fusion by destabilizing gB. Mutations in gB that obscure the pocket reduce fusion. Mutation of gH-V831 also reduces fusion. If the gH tail is modeled as an extended peptide, gH-V831 lies the same distance from the membrane as this pocket. Unexpected results that some of the gB pocket mutations do not reduce fusion are described forthrightly and the explanation is plausible (that the mutations both obscure the pocket and destabilize the protomer contacts). The work provides a functional explanation for newly and previously identified hyperfusogenic mutations in the gB tail that do not directly disrupt interprotomer contacts (the rim mutants).

The proposed model is clear and supported by carefully considered results. The manuscript is very well written, placing the work in the context of previous studies. The work provides mechanistic explanation of herpesvirus entry, generating a valuable model that is relevant to all herpesviruses and to other viral fusogens.

**Minor Issues: Editorial and Data Presentation Modifications**

Page 1, line 3: Consider removing the word "unusually" to describe control of fusion by the cytoplasmic tail of the fusion protein. HIV and paramyxovirus fusion proteins are also influenced by their cytoplasmic tails.

**Response:** We thank the reviewer for pointing this out and have made the suggested change. We have also added references to HIV and paramyxoviruses within the introduction on lines 89-91.

Page 6, line 19: Clarify what "impairs fusion rather than refolding" means. Does this statement mean simply that A851V was not misfolded or does it mean that the mutant does not affect gB refolding? I believe this sentence means that A851V is the first properly folded hypofusogenic gBCTD mutant, but I wasn't sure.

**Response:** We apologize for the lack of clarity. We meant to state that A851V is properly folded and hypofusogenic. We have modified the text on lines 162-164 accordingly.

Page 10, line 16 and Figure 5b: Clarify the concept of the "fault line". Does "fault line" simply mean the interface between adjacent protomers near this pocket that is disrupted to trigger fusion?

**Response:** We have replaced “fault line” with “protomeric interface” on line 256.

Fig. 4e: Does the horizontal line for statistical comparison apply only to V831T? If so, consider drawing the line as in Fig. 4f, where the vertical lines do not cross the horizontal lines. The current Fig. 4e could be interpreted to mean that all the mutants under the line have p <0.05.

**Response:** We have changed the line in Fig. 4e to match the one in Fig. 4f to clarify that indeed only V831T is statistically significant.

Brief additions to the discussion:

If the authors know whether the gB hyperfusogenic mutants (the new A851 mutants or relevant previously characterized mutants) retain gH/gL-dependence for fusion, they could discuss why mutations in the gB pocket or rim alone are insufficient to trigger gB. This is not a request for additional experiments, as the findings are not critical for the conclusions of this work.

**Response:** This important point was also raised by Reviewer 1. We and others have shown that the hyperfusogenic mutants of HSV-1 gB still require gH/gL, which suggests that these mutations weaken the gB_CTD_ clamp but do not abolish it. We have added additional explanation in that paragraph, lines 503-507, to clarify that we hypothesize that the A851F, A851W, A851E, and A851K mutants act similarly to other hyperfusogenic mutants containing mutations designed to destabilize the clamp.

In the 'open questions' section, the authors could comment on whether they expect that the ectodomains of gB and gH/gL also interact (in addition to the tails).

**Response:** We investigated this important question in a recent study that is currently under peer review and has been uploaded to BioRxiv (doi: https://doi.org/10.1101/2022.05.07.491029). Specifically, we found that gH and gB interact through their ectodomains, transmembrane domains, and cytoplasmic domains, although the respective binding sites have not yet been mapped. We have added text in the Discussion on lines 555-558 to highlight these findings.

In the 'open questions' section, the authors could comment on how they anticipate that gH-V831 regulates fusion, since gH-V831 does not act as a wedge for gB until signaled by a receptor-binding protein interacting with gH/gL (presumably in the ectodomain).

**Response:** Thank you for this suggestion. We have added text in the Discussion on lines 560-565 to explain our hypothesis for what triggers the wedging action of gH V831.
